# Partial coherence boosts photonic computing

**DOI:** 10.1038/s41377-024-01571-6

**Published:** 2024-08-29

**Authors:** Hang Chen, Fei Dai

**Affiliations:** 1 Department of Electronic Engineering, Tsinghua University, Beijing, China; 2 School of Electronic and Information Engineering, Beihang University, Beijing, China

**Keywords:** Optics and photonics, Optical physics

*Nature* **632**, 55–62 (2024)

10.1038/s41586-024-07590-y

The advent of coherent lasers has been a cornerstone of modern optics, with the prevailing belief that greater coherence always confers an advantage. However, researchers from the University of Oxford, A*STAR in Singapore, Heidelberg University, the University of Münster, and Ghent University have developed an innovative approach that challenges this conventional wisdom. They introduced a partially coherent photonic convolutional processing system. Within a specified bandwidth, partially coherent light remains unaffected by phase fluctuations, enabling improved photonic computing parallelism through wavelength-division multiplexing without significantly compromising system performance. Additionally, this technique substantially reduces the feedback control and thermal management demands present in coherent systems, thus enhancing the practical application of high-throughput photonic computing. Notably, this method exhibited exceptional performance in gait classification tasks for Parkinson’s disease patients and handwritten digit classification tasks, underscoring the potential of partial coherence to tackle increasingly complex computing challenges.

